# Knockdown of mental disorder susceptibility genes disrupts neuronal network physiology *in vitro*

**DOI:** 10.1016/j.mcn.2010.12.014

**Published:** 2011-06

**Authors:** Erik J. MacLaren, Paul Charlesworth, Marcelo P. Coba, Seth G.N. Grant

**Affiliations:** Genes to Cognition Programme, Wellcome Trust Sanger Institute, Hinxton, Cambridge CB10 1SA, UK

**Keywords:** Multielectrode array, Neuronal culture, Tnik, Dlg2, Disc1, Dctn5

## Abstract

Schizophrenia and bipolar disorder are common diseases caused by multiple genes that disrupt brain circuits. While great progress has been made in identifying schizophrenia susceptibility genes, these studies have left two major unanswered mechanistic questions: is there a core biochemical mechanism that these genes regulate, and what are the electrophysiological consequences of the altered gene expression? Because clinical studies implicate abnormalities in neuronal networks, we developed a system for studying the neurophysiology of neuronal networks *in vitro* where the role of candidate disease genes can be rapidly assayed. Using this system we focused on three postsynaptic proteins *DISC1*, *TNIK* and *PSD-93*/*DLG2* each of which is encoded by a schizophrenia susceptibility gene. We also examined the utility of this assay system in bipolar disorder (BD), which has a strong genetic overlap with schizophrenia, by examining the bipolar disorder susceptibility gene *Dctn5*. The global neuronal network firing behavior of primary cultures of mouse hippocampus neurons was examined on multi-electrode arrays (MEAs) and genes of interest were knocked down using RNAi interference. Measurement of multiple neural network parameters demonstrated phenotypes for these genes compared with controls. Moreover, the different genes disrupted network properties and showed distinct and overlapping effects. These data show multiple susceptibility genes for complex psychiatric disorders, regulate neural network physiology and demonstrate a new assay system with wide application.

## Introduction

Schizophrenia is a psychiatric disorder with a high heritability that is not caused by a common single gene mutation but rather by dozens or perhaps hundreds of mutations including both single nucleotide polymorphisms (SNPs) in coding and regulatory regions as well as copy number variants (CNVs) (Craddock et al., 2005, Stone et al., 2008). While this disparate set of mutations is involved in producing similar behavioral effects in patients, the identity of the genes has not uncovered a single family or category of molecules but rather has broadly implicated biological processes such as neurodevelopment and synaptic transmission. While it may be implied that these genes all impinge on some common aspect of neuronal and brain function, there is still a lack of neurophysiological methods for testing and comparing many genes in many different functional classes which would be required to tease out the differential roles each susceptibility gene plays in the etiology of schizophrenia.

There is evidence to support the hypothesis that schizophrenia is a disorder of brain circuits (Arnold et al., 2005) and recordings from patients with schizophrenia, bipolar disorder (BD) and autism show abnormalities in patterns of neural activity (Adler et al., 2006; Ford et al., 2007; Uhlhaas and Singer, 2007). More recently, synchrony in the activity between neurons of the hippocampus and prefrontal cortex was found to be impaired in a model of schizophrenia in which a human microdelection in chromosome 22 (22q11.2) was reconstituted in a mouse (Sigurdsson et al., 2010). This study demonstrated a predictive correlation between the degree of neuronal synchrony and the ability of the animal to perform a learning task. Although none of the genes examined in this work is in the 22q11.2 region, this study suggests the possibility that *in vitro* cellular deficits can be a proxy, or indicator, for *in vivo* behavioral phenotypes.

Primary cultures of hippocampal neurons from embryonic mice grown on multielectrode arrays demonstrate behavioral characteristics such as bursting and synchronized firing, and could provide a model system for monitoring and measuring the effects of genetic manipulations on the development of neural networks. More specifically, these cultures form networks that spontaneously generate action potentials (spikes), rapid bursts of spikes and synchronized firing, which become increasingly coordinated in spatial and temporal dimensions as the cultures mature (Chiappalone et al., 2006). This network activity can be readily recorded using MEAs (Valor et al., 2007) which allow the network-level effects of any manipulation of the cultures to be observed. Since many candidate mutations for schizophrenia and related psychiatric disorders involve a loss of the gene product, either through a deletion of some or all of the exons or a translocation event that interrupts the genomic structure of the gene, RNA interference (RNAi) offers a simple, rapid and flexible method of modeling the effect of these mutations directly in primary cultures. Previous studies have documented the effects of pharmacological interventions on the network properties of cultured neurons (Arnold et al., 2005; Gramowski et al., 2004), however the contributions of targeted gene knockdown, including disease gene candidates, to these phenotypes have not yet been reported.

In order to determine if common network deficits were created by schizophrenia-related genes, we studied the impact of gene knockdown on neuronal networks grown on MEAs using siRNAs and targeted three genes associated with schizophrenia: TRAF2 and NCK interacting kinase (*Tnik*), Discs Large Homolog 2 (*Dlg2*/*PSD-93*), and Disrupted in Schizophrenia 1 (*Disc1*), as well as a candidate gene for bipolar disorder (BD), Dynactin 5 (*Dctn5*). All of these genes have either been localized to the post-synaptic density (PSD) directly or, as in the case of *Dctn5*, are part of a multiprotein complex that has been found at the PSD (DeGiorgis et al., 2006; Kirkpatrick et al., 2006; Kwinter et al., 2009). *Tnik* (TNIK) is a kinase that localizes to the post-synaptic density and interacts with other proteins strongly associated with schizophrenia including NMDA receptors and DISC1 (Wang et al., 2010). *Dlg2* (PSD-93/Chapsyn-110) is a scaffolding protein of the post-synaptic density deleted in schizophrenia in a study of Genome-Wide Copy Number Variation (Walsh et al., 2008) and shows a reduction in protein expression in post-mortem brain samples from schizophrenics (Kristiansen et al., 2006). Moreover, mouse knockouts of Dlg2 show hypofunction of NMDA receptor signaling (Carlisle et al., 2008), a process implicated in schizophrenia (Javitt, 2007; Mohn et al., 1999). A hemizygous translocation that disrupts the *Disc1* gene segregates with schizophrenia and other psychiatric disorders in a Scottish pedigree (Millar et al., 2000). Finally, schizophrenia and BD likely share common genetic mechanisms (Purcell et al., 2009) and so we also tested a BD susceptibility gene, Dynactin 5. *Dctn5* (p25) encodes a subunit of the dynactin/dynein motor complex, which is known to be important for retrograde dendritic transport in neurons, and lies within a genomic region that has been identified by a Genome-Wide Association Study (GWAS) in BD (Burton et al., 2007). Furthermore, components of the dynactin complex have previously been associated with motor neuron degeneration and familial dementia in animals and humans (Laird et al., 2008; Munch et al., 2005).

## Results

**Fig. 1:**
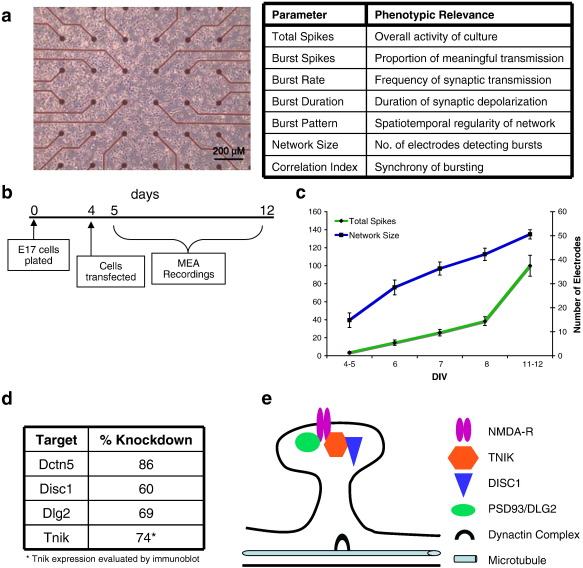
*Experimental strategy.* (a) A phase-contrast image of WT neurons grown on an MEA at 4 DIV. Scale bar is 200 μm. The table lists the seven network parameters quantified using MEA recordings. (b) Experimental design of study. Cultures were transfected at 4 DIV and recorded for 15 min daily until 12 DIV. (c) In control WT cultures, the total spikes and network size increase from 4 DIV to 12 DIV as the cultures mature. (d) Level of knockdown achieved as determined by real-time PCR or Western blot. In all cases, the level of target gene expression is significantly reduced compared to both untransfected and NTC cultures. The untransfected and NTC cultures did not demonstrate any differential expression of these genes compared to one another. *p < 0.05. (e) Model showing the synaptic localization of all the genes examined.

Hippocampal neurons from E17.5 mouse embryos were cultured on MEAs (Fig. 1a) and transfected after 4 days *in vitro* (DIV 4). The four target genes were tested using three different conditions: transfection of siRNAs targeting the gene of interest and two negative controls, untransfected controls and transfection of non-targeting siRNA (NTC). The network activity of the neurons was recorded for 15 min daily beginning on DIV 5 and continuing until DIV 12 (Fig. 1b). Knockdown for all genes was assayed at DIV 7 (72 h post-transfection) and ranged from 60 to 84% compared with control cultures (Fig. 1d), which is in the range relevant to the levels of expression occurring in the human hemizygous mutations (Kristiansen et al., 2006; Millar et al., 2005). Because the mRNA and protein were harvested from the entire culture, and are derived both from neurons transfected with the siRNAs and untransfected cells, the level of expression knockdown can be taken as a lower limit of the transfection efficiency. That is, if the siRNA pool targeting *Dctn5* reduces mRNA expression by 85% overall in the culture, 85% of the cells in that culture have been transfected at a minimum. Thus, the siRNAs used to target the genes in this study are transfected into the majority of the cells in the cultures.

During the first 12 days in control cultures, a dramatic transition from silent neurons to active synchronized cultures was observed as an increase in both the number of spikes recorded each day as well as an increase in the number of electrodes recording bursts (Fig. 1c). The MEA recordings allowed quantitation of the following seven parameters of neural network activity that were used to observe the phenotypic effects of the gene knockdowns: i) total spikes, ii) % of spikes in bursts, iii) burst rate, iv) burst duration, v) burst pattern, vi) network size and vii) correlation index (summarized in Fig. 1a). These seven parameters have a high degree of information content describing the activity of the developing neuronal networks, and allow the effect of an siRNA to be determined in multiple dimensions and therefore facilitate the direct comparison of genes with disparate cellular functions. A summary “barcode” of the knockdown results is presented in Fig. 3 showing parameters that are significantly increased or decreased by the knockdown of each gene at each DIV measured. In addition to this summary barcode, the detailed measurements for each parameter are reported for each of the five timepoints in culture (Supplementary Tables 2–5).

In order to check for evidence of significant toxicity of the siRNA in the cultures, the total spike parameter was examined first. The total number of spikes detected on MEAs depends, in part, on the number of cells in close proximity to the electrodes (Wagenaar et al., 2006) as well as the synapse density in the culture (Brewer et al., 2009). Total spikes can therefore be used as an indirect measure of toxicity with a decrease in the overall number of spikes suggesting a toxic effect of the siRNAs. There was no significant difference in total spikes, neither an increase nor decrease, recorded between transfected and non-transfected cultures under any of the conditions examined (*p* > 0.05) (Supplementary Fig. 1), nor any detectable loss of neurons on visual inspection. In the absence of obvious cytotoxic effects, the bursting behavior in the siRNA treated networks was next examined in more detail.

**Fig. 2:**
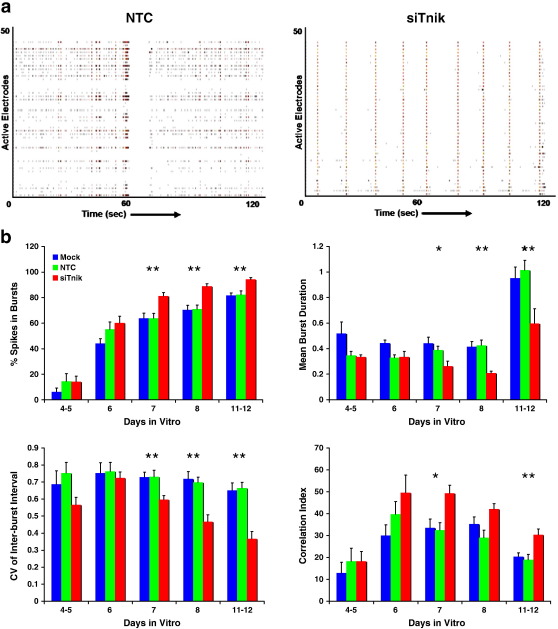
*Tnik knockdown affects network phenotypes.* (a) Raster plots of network firing recorded on DIV 7 in an untransfected and *Tnik* knockdown culture showing 2 min of activity across all active electrodes. Each horizontal row represents one electrode on the MEA and each tic-mark represents a spike detected by the electrode. Closely packed tics, such as those that are boxed represent bursts. (b) Percentage of spikes in bursts (Burst Spikes) is increased while the burst rate is reduced in cultures transfected with siRNAs targeting *Tnik* as represented by the red bars. Additionally, the burst pattern parameter is reduced and the correlation index is increased, both indicative of increased synchrony in network bursting. Untransfected and NTC treated cultures are shown in blue and green bars respectively, while *Tnik* knockdowns are plotted in red. Mean data are plotted (±SEM). The data were analyzed by ANOVA and Fisher's PLSD post-hoc tests. *p <  0.05 and **p < 0.01.

The network effects of knocking down *Tnik* in these cultures were the most dramatic as shown in Fig. 2. The effect was dramatic enough to be observed by raster plot of the firing frequency recorded by the electrodes of the MEAs (Fig. 2a). The significantly altered parameters included: increased percentage of spikes in bursts, decreased duration of bursts and increased coordination of bursting activity across neurons in the network as reflected by the decreased burst pattern and increased correlation index parameters. All quantitative data is shown in Supplementary Table 2. The burst correlation in particular is of interest because of the connection between schizophrenia and neural network synchrony. In all cases the effect observed becomes significant only after 7 DIV, indicating that the networks remained normal for at least three days after application of the siRNA, and remained significant through the 12th day of culture. The delayed onset of effect after the application of the siRNA could be due to the time it takes for protein to turnover in the cell after new translation has stopped. The 11–12 DIV timepoint is past the expected efficacy of siRNA treatment, and could also suggest the protein turnover “lag” between translation and function. However, it may also indicate irreversible effects to the development of neuronal networks that cannot be compensated at a later time.

**Fig. 3:**
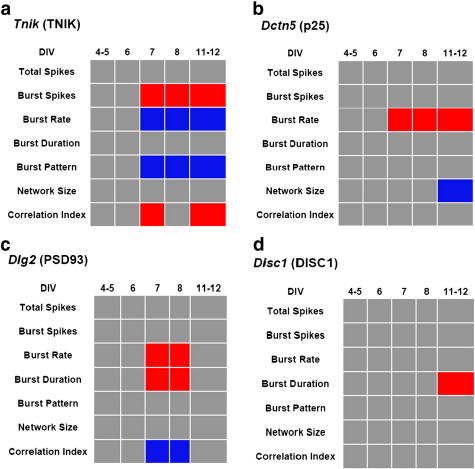
*Summary of results for all genes tested.* Red indicates a significant increase and blue a significant decrease (*p* < 0.05) of that parameter at that timepoint *versus* untransfected and NTC treated cultures and no significant difference between untransfected and NTC (*p* > 0.05). Gray indicates no significant difference. Significance was determined by ANOVA and Fisher's PLSD post-hoc tests.

In parallel experiments, *Dlg2* mRNA levels were reduced in the neuronal cultures by 69 ± 3% (*p* < 0.01) with siRNA treatment and, most strikingly, caused an increase in the observed bursting rate (Fig. 3c and Supplementary Table 4). We also observed an increase in the average duration of bursts, in contrast to the *Tnik* results. These differences in burst rate and duration disappeared by one week post-transfection (DIV 12), returning to the same levels as the controls (Fig. 3c). This suggests a transient effect of the siRNA from which the cultures later recover, a marked difference from the *Tnik* results. Thus *Dlg2* was also required for normal neuronal network activity.

RNAi knockdown of *Disc1* resulted in minimal phenotypic effects compared with the other two genes, with only an increase burst duration reaching statistical significance at DIV 12 (Fig. 3d and Supplementary Table 5). While it remains possible that the 60 ± 19% (*p* < 0.05) reduction in mRNA was insufficient to produce a more robust phenotype, this decrease is similar to the 40–50% reduction of mRNA reported in a human study (Millar et al., 2005).

Lastly, we targeted *Dctn5*, a BP risk gene, for knockdown and succeeded in reducing its expression by 86 ± 2% (*p* < 0.01). The most striking finding was that the rate of bursting was elevated to 5.8 bursts per minute in *Dctn5* knockdowns (*p* < 0.01) with a similar number of bursting electrodes (Fig. 3). This increase in burst rate was observed with *Dctn5* knockdown three days after transfection (DIV 7) and persisted until the end of the recording period (DIV 12; Fig. 3). Also, in contrast to the control cultures that show a steady increase in the network size over the recording period, *Dctn5* knockdown prevented this growth from DIV 6 onward, a difference that becomes significant by DIV 12.

## Discussion

The genes assessed in this study are associated with two diseases, schizophrenia and BD, that are known from GWAS studies to have common genetic underpinnings (Purcell et al., 2009). The genes themselves are also closely associated with the same subcellular compartment in the neuron, namely the post-synaptic density (PSD) and might therefore be expected to affect network function through multiple, partially overlapping effects on the synapse. Considering that these diseases are thought to arise from neurodevelopmental mechanisms with electrophysiological consequences (Blumberg et al., 2004; Lewis and Levitt, 2002), the MEA system described here has potential to measure the effects of susceptibility genes, if indirectly, at the biological level of network function. Moreover, since the effects observed in this study involve networks of many neurons rather than individual cells, the results may be considered more indicative of the disease state in an intact brain and thus more relevant to the neural synchrony hypotheses that have been proposed such as that of hippocampal hyperactivity in schizophrenia discussed below.

These results indicate that knockdown of three susceptibility genes, *Tnik*, *Dlg2* and *Dctn5*, results in abnormal hyper-activity and disrupted development of neural networks *in vitro*. Moreover, the utility of this assay of neuronal genetic manipulations derives from the fact that overall network activity is a higher-order measure of neural physiology *in vitro* than more reductive assays. It should be remembered as a caveat to these findings that the level of knockdown for each gene at the mRNA level was different, ranging from 85% overall for *Dctn5* to as little as 60% for *Disc1*. Another caveat of this method using transient transfections of siRNA duplexes is that full knockdown effects are only achieved for a short period, typically lasting less than one week. We have here reported phenotypic effects in the cases of *Tnik*, *Disc1* and *Dctn5* knockdown at 12 DIV, a full eight days after the transfection of siRNAs. This result could indicate a slow protein turnover at the synapse, or alternatively, a role for these three genes in neural network development (at least in cultures). *Dlg2*, in contrast, displayed acute knockdown effects on network firing that recovered by the end of the recording period. This is more consistent with a role in the ongoing synaptic function, rather than development.

The most consistent result across all of the knockdown experiments conducted with these susceptibility genes was an increase in either bursting rate, duration and/or pattern. The increases observed in these parameters fit well with the hypothesis of hyperactive hippocampal function underlying schizophrenic disregulation postulated by Anthony Grace and others (Lodge and Grace, 2007; Krieckhaus et al., 1992). Clearly the hippocampus is not the only region disrupted by schizophrenia and BD, however as a pilot case for using this experimental paradigm, the fact that we observe the same hyperactivity of hippocampal neurons observed *in vivo* helps to validate the approach.

To our knowledge, there is no published data showing that schizophrenia and BD susceptibility genes interfere with neuronal network function. Our findings suggest that this neurophysiological impairment may be indicative of many psychiatric susceptibility genes. Of further interest is the distinct yet overlapping nature of the network changes caused by the downregulation of the different genes. In particular the role of *Tnik* in the correlation of network firing echoes the findings of other groups in neural circuit synchrony. Also intriguing is the fact that *Dctn5* and *Dlg2* produce a convergent phenotypic effect on the burst rate despite having completely different functional roles in the neuron. These findings suggest that it will be of considerable interest to test many other candidate genes and map the converging phenotypes in greater detail, and thereby compare more psychiatric disease genes. In this way a larger “interactome” of neuronal gene functions might be defined to help understand the wide array of genes underlying neural disease.

This work supports the hypothesis that disorganization of neuronal networks may be a simple and reliable indicator to test the many genes that are thought to predispose individuals to schizophrenia and BD as well as other psychiatric diseases. The MEA assay system is amenable to drug intervention, stem cell derived neurons and potential scaling to high-throughput to the level of genome-wide analysis. The development of novel neurophysiological methods is an important requirement for this era of genomic medicine.

## Experimental methods

### Primary neuronal cultures

*Dctn5* knockdown and control experiments utilized neurons derived from both C57BL/6-*Tyr*^*c-Brd*^ and 129S5/SvEvBrd strains of mice as cultures derived from these strains show no differences in the reported parameters (unpublished data). Knockdown and control cultures for *Tnik*, *Dlg2* and *Disc1* included neurons from the C57BL/6-*Tyr*^*c-Brd*^ strain only. All mice were treated in accordance with the UK Animals Scientific Procedures Act of 1986, and all procedures were approved through the UK Home Office Inspectorate. The hippocampus and cortex were dissected from E17.5 mouse embryos and transferred to papain (10 units/mL Worthington, Lakewood, NJ) for 22 min at 37 °C. Cells were manually dispersed in Dulbecco's Phosphate-Buffered Saline (Invitrogen, Carlsbad, CA) and centrifuged twice at 400 ×*g* for 3.5 min. The final pellet was resuspended in Neurobasal/B27 supplemented with 0.5 mM Gln (Invitrogen), and dissociated cells were plated onto poly-d-lysine and laminin-coated substrates at a density of 1500 cells/mm^2^. Hippocampus neurons were plated onto MEAs for electrophysiology and cortical neurons were plated on plastic culture dishes for total RNA extraction. One third of the culture medium was changed every 3–4 days throughout the experiment.

### siRNA transfection

Once cultures reached 4 DIV, they were transfected with pools of siRNAs. The siRNAs used were either non-targeting siGLO green siRNAs (cat# D-001630-01-05) or pools consisting of four separate siRNA duplexes targeting *Tnik* (cat# L-167234-00), *Dlg2* (cat# L-043520-00), *Disc1* (cat# L-054060-01), or *Dctn5* (cat# L-063270-01, Dharmacon, Lafayette, CO) and were transfected according to the manufacturer's instructions. Briefly, the siRNAs and transfection reagent were incubated separately with unsupplemented NeuroBasal medium at room temperature for 5 min. Next the diluted siRNAs and transfection reagent were combined and incubated at room temperature for a further 20 min before being diluted to a 3× concentration with NeuroBasal medium supplemented with B-27 and l-glutamine. The mix was added to the culture medium to result in a final concentration of 100 nM siRNA with 2.4 μL of DharmaFECT-3 transfection reagent (Dharmacon) in a total culture volume of 600 μL. Untransfected control cultures had a one-third volume medium change only.

### RNA isolation and real-time RT-PCR

Cells were lysed and total RNA was extracted at DIV 7 using RNeasy Mini Kits and columns and an on-column DNase treatment was carried out to remove DNA with RNase-free DNase (Qiagen, Valencia, CA) following the manufacturer's instructions. Total RNA was eluted in 30 μL H_2_O and the quantity and purity were checked using an ND-1000 spectrophotometer (NanoDrop, Wilmington, DE). Next, 1 μg of total RNA was used for the reverse transcription with SuperScript III Reverse Transcriptase (Invitrogen) following the manufacturer's protocol.

Real-time PCR reactions were set up using 1 μL of cDNA and primers at 300 nM in 20 μL of 1× Quantitect SYBR Green PCR Master Mix (Qiagen). The sequences of the primers are provided in Supplementary Table 1. Reactions were cycled in the 7500 Real-Time PCR System (Applied Biosystems, Foster City, CA) with the following conditions: 1 cycle of 95 °C for 15 min, then 40 cycles of 95 °C for 15 s and 60 °C for 1 min followed by a dissociation cycle. The data generated were analyzed with the 7500 System SDS version 1.2.2 software using the standard curve method. *Gapdh* was used as an endogenous control. Reactions for each pair of primers were run at least in triplicates. ANOVA and Fisher's PLSD post-hoc test were used to statistically analyze the quantitative data.

### Immunoblot

Tnik protein levels were determined by Western blot. Standard procedures were used for protein extraction, SDS-PAGE, and Western blotting (Komiyama et al., 2002). The primary antibody against Tnik protein was used at a concentration of 1 μg/mL (BD Biosciences, Franklin Lakes, NJ). The blots were quantified in a Kodak Image station 4000MM and analyzed with the Kodak molecular image software 4.0.

### Network data collection and analysis

Cells were plated directly onto coated MEAs that consisted of 8 · 8 grids of titanium nitride electrodes of 30 μm diameter and interelectrode spacing of 200 μm (MultiChannel Systems, Reutlingen, Germany). Pairs of MEAs were interfaced with duplex 60 channel amplifiers (MultiChannel Systems) and spontaneous activity was recorded in 15 min epochs daily in the same NeuroBasal medium used to maintain the cultures. Spontaneous spikes were detected and analyzed by using MC Rack (MultiChannel Systems) and action potentials were digitized with a 128-channel analog/digital converter card by sampling the 1 ms pre- and 2 ms post-crossing of threshold at a rate of 25 kHz. Detection thresholds were set to a fixed level of − 20 μV, approximately 6–8 standard deviations of baseline noise level. Timestamps were extracted using batch scripts written for NeuroExplorer (Nex Technologies, Littleton, MA) and analyzed using custom-written software developed in the R statistical programming environment (www.r-project.org) to compute parameters that quantitatively describe network activity. A burst-detection algorithm similar to the “max interval method” used in NeuroExplorer was implemented to classify trains of action potentials with these characteristics as bursts. This method parses a spike train into bursts based on various thresholds for the interspike interval (ISI) between spikes starting and ending a burst, plus thresholds for deciding when to merge bursts. ANOVA and Fisher's PLSD post-hoc test were used to statistically analyze the quantitative data. Calculation of the individual parameters was done as follows:

*Total spikes*: The sum of all spikes detected in a 15 min recording epoch.
*% of spikes in burst*: Percentage of spikes fired within bursts.
*Burst rate*: Average bursts per minute across all electrodes detecting more than one burst per minute.
*Burst duration*: Average duration of bursts detected at electrodes detecting more than one burst per minute.
*Burst pattern*: The coefficient of variation of the inter-burst interval. The intervals between bursts of spikes are averaged across the whole recording for each electrode and a coefficient of variation is calculated from these values. Higher values reflect a lack of temporal structure to activity and lower values indicate a greater degree of temporal organization.
*Network size*: The number of electrodes detecting at least one burst per minute. This reflects the size of the network since only cells that are part of the network are likely to generate bursts of spikes regularly throughout the recording session.
*Correlation index*: The degree of correlation of burst detection between pairs of electrodes using the method of Wong et al. (1993) and a bin width of 0.1 ms.

The following are the supplementary materials related to this article.

Supplementary Table 1 Sequences of primers used in real-time RT-PCR experiments.

Supplementary Table 2 Full dataset for *Tnik* knockdown cultures.

Supplementary Table 3 Full dataset for *Dctn5* knockdown cultures.

Supplementary Table 4 Full dataset for *Dlg2* knockdown cultures.

Supplementary Table 5 Full dataset for *Disc1* knockdown cultures.

Supplementary Fig. 1 Western blot of *Tnik* knockdown in culture. The blots were scanned in a Kodak Image station 4000MM and quantitated with Kodak molecular image software 4.0.

Supplementary Fig. 2 The total spike parameter is not significantly affected by knocking down *Tnik* (a), *Dctn5* (b), *Dlg2* (c) or *Disc1* (d), implying a lack of toxicity due to transfection or RNAi effects. ANOVA (*p* > 0.05).

## Financial disclosures

EJM, PC, MC and SGNG reported no potential conflicts of interest.
