# Supplementary figures and images for: Knockdown of mental disorder susceptibility genes disrupts neuronal network physiology in vitro
Source: Mol Cell Neurosci. 2011 Jun;47(2):93–9. doi: 10.1016/j.mcn.2010.12.014 (PMC3105225; doi:10.1016/j.mcn.2010.12.014)

## Slide 1
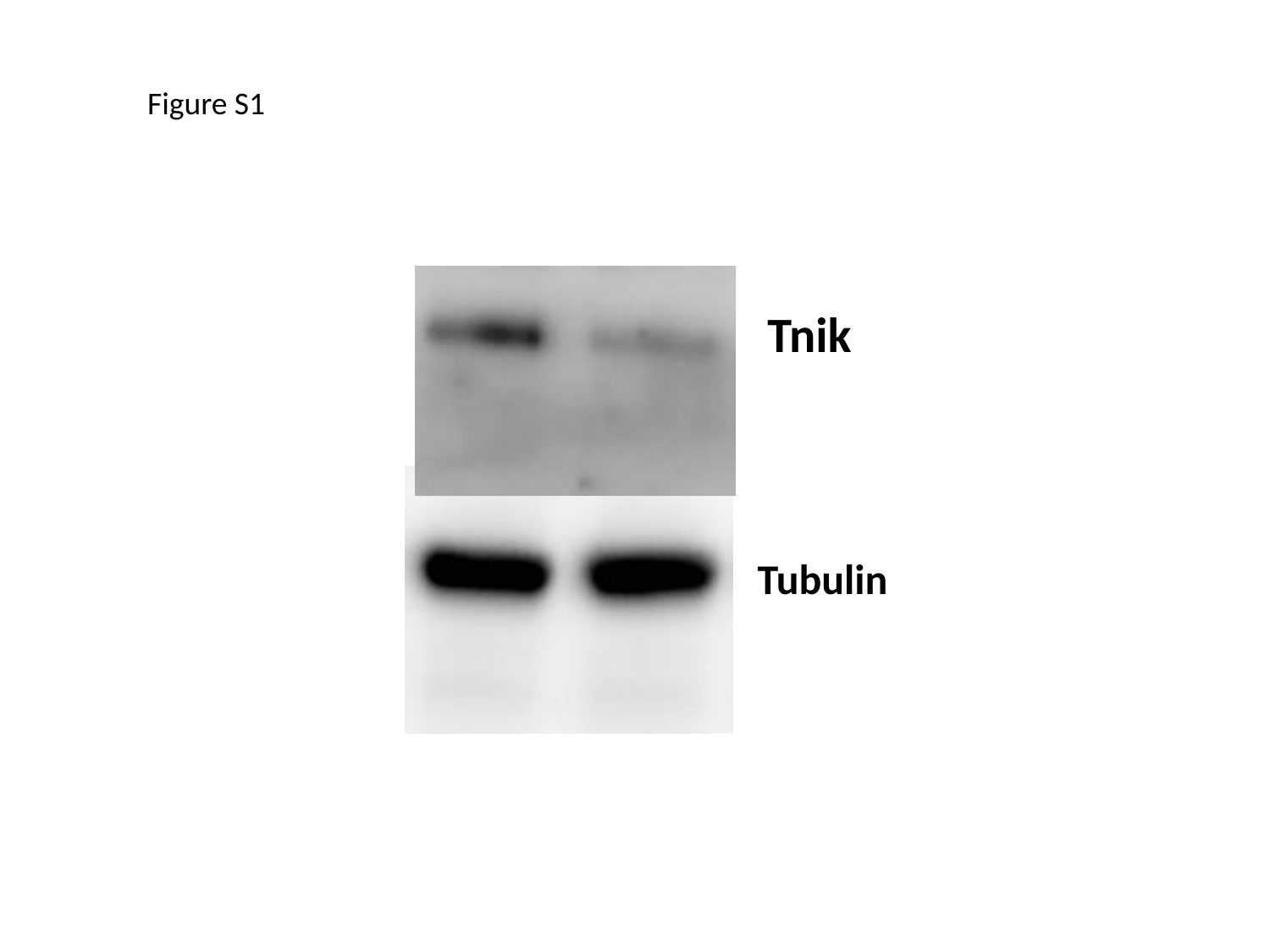

Figure S1
Tnik
Tubulin

Supplement: Supplementary Fig. 1 — Western blot of Tnik knockdown in culture. The blots were scanned in a Kodak Image station 4000MM and quantitated with Kodak molecular image software 4.0. [file mmc6.ppt]

## Slide 1
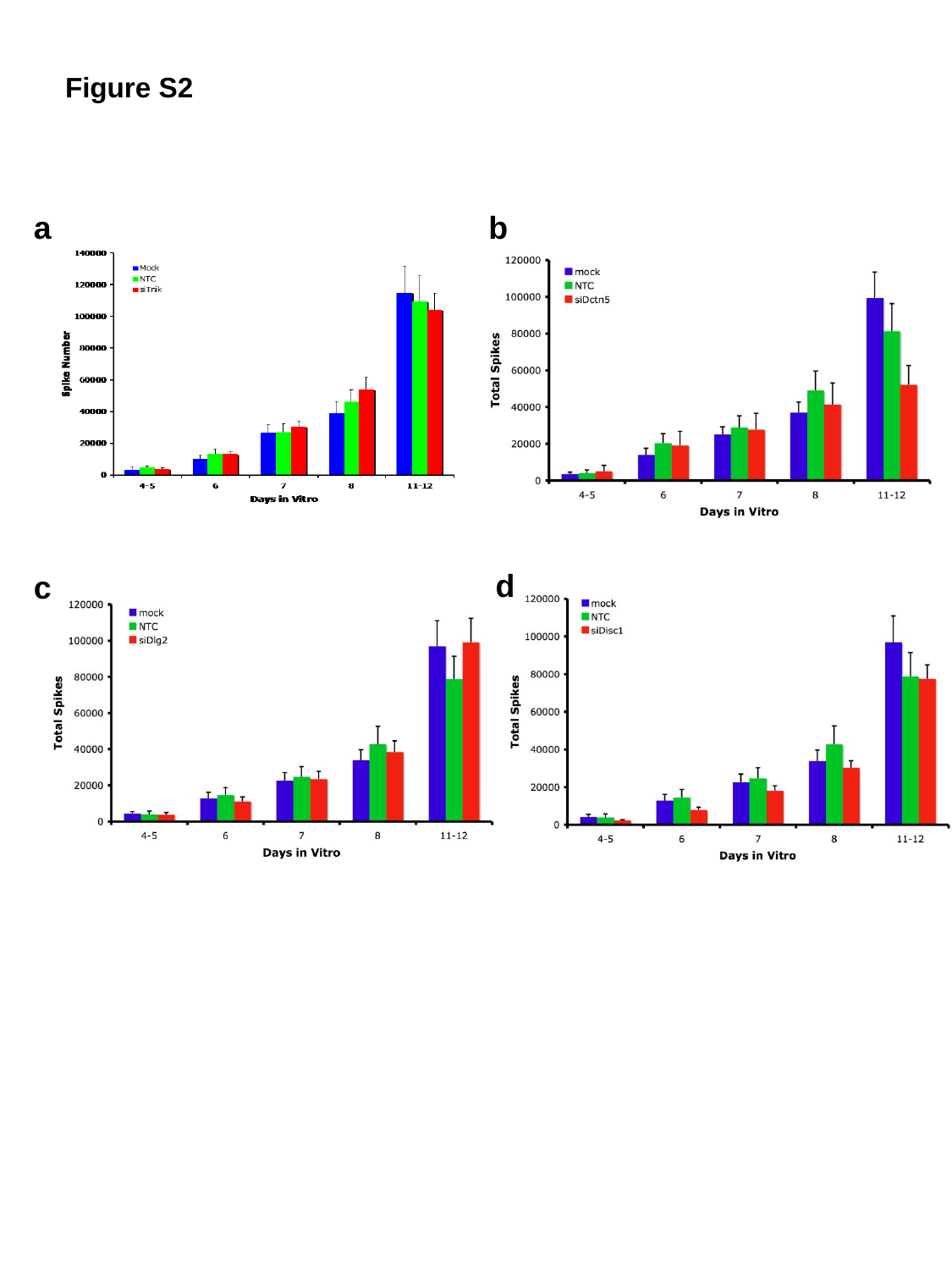

Figure S2
a
b
d
c

Supplement: Supplementary Fig. 2 — The total spike parameter is not significantly affected by knocking down Tnik (a), Dctn5 (b), Dlg2 (c) or Disc1 (d), implying a lack of toxicity due to transfection or RNAi effects. ANOVA (p > 0.05). [file mmc7.ppt]
